# What transgender individuals in Japan expect from gender‐affirming surgery: A multicenter prospective observational study

**DOI:** 10.1002/pcn5.70207

**Published:** 2025-09-19

**Authors:** Wakako Yorozuya, Koji Ichihara, Nodoka Kozen, Teruo Abe, Manabu Nakagawa, Koji Niwa, Tsuyoshi Baba, Utako Ikeda, Satoshi Nishikawa, Hiroshi Ikeda, Toshiaki Endo, Azusa Yamana, Daito Nishiyama, Naoya Masumori

**Affiliations:** ^1^ Department of Urology Sapporo Medical University School of Medicine Sapporo Hokkaido Japan; ^2^ Department of Urology Sapporo Central Hospital Sapporo Hokkaido Japan; ^3^ Abe Mental Clinic Urayasu Chiba Japan; ^4^ Nagumo Clinic Osaka Osaka Japan; ^5^ Graduate School of Science and Technology Keio University Minato Tokyo Japan; ^6^ Department of Obstetrics and Gynecology Sapporo Medical University School of Medicine Sapporo Hokkaido Japan; ^7^ Miyanomori Ladies' Clinic Sapporo Hokkaido Japan; ^8^ Rise Maruyama Clinic Sapporo Hokkaido Japan; ^9^ Medical Corporation Hokujinkai Miki Mental Clinic Sapporo Hokkaido Japan; ^10^ Women's Medical Center, Tokeidai Memorial Hospital Sapporo Hokkaido Japan

**Keywords:** gender incongruence, gender‐affirming surgery, sex reassignment surgery, sterilization surgery, transgender

## Abstract

**Aim:**

To understand the preferences of individuals with gender incongruence (GI) regarding gender‐affirming surgery (GAS), including gonadectomy.

**Methods:**

A prospective, multicenter survey was conducted in Japan targeting individuals aged 18 years or older with GI who had not yet undergone GAS. Participants completed a questionnaire about their GAS preferences, desired surgical procedures, the reasons for their preferences, and willingness to undergo gonadectomy even if it was not legally required.

**Results:**

In total, 107 participants (82 assigned female at birth [AFAB] and 25 assigned male at birth [AMAB]; median age: 31) participated in the study. Of those, 69% desired GAS, 29% did not, and 2% were undecided. The primary reasons for desiring GAS were the need for legal gender change (positive feelings: 62%, unavoidable: 24%), mental stability (56%), and reducing gender dysphoria (54%). Conversely, the main reasons for not wanting GAS were difficulty in securing funds for surgery (58%), followed by resistance to gonadectomy (41%). Of the 53 AFAB participants, 52 individuals desired hysterectomy and oophorectomy, and 26 wanted only this surgery. Among the 22 AMAB participants, 13 individuals desired vaginoplasty; however, five withdrew due to concerns about costs or postoperative complications. Even without legal requirements, 47% said they would choose gonadectomy.

**Conclusion:**

Reforming legal requirements for gonadectomy may alter the demand for or details of GAS in Japan, yet half of the participants still desire surgery. The primary reason for not undergoing surgery is the difficulty in securing funds, which is a significant finding.

## Graphical abstract

Preferences for gender‐affirming surgery in Japan were shaped by legal and psychological factors, with financial difficulty as the main barrier.

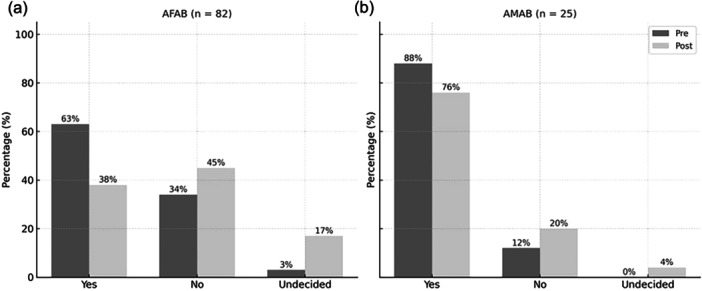

## INTRODUCTION

Transgender healthcare in Japan was standardized in 1997 with the establishment of the “Guidelines for the Diagnosis and Treatment of Gender Identity Disorder.”^1^ The fifth revised edition is currently in effect as of August 2024.^2^ The current guidelines reflect the diagnostic criteria and classification of the DSM‐5^3^ and ICD‐11.^4^ What was previously referred to as “gender identity disorder (GID)” is now defined as “gender incongruence (GI),” a “condition related to sexual health,” and has been de‐pathologized. Since 1998, gender‐affirming surgery (GAS) has been performed as a legitimate medical procedure under the guidelines. Since 2018, mastectomy (chest wall masculinization) and GAS have been covered by health insurance. However, gender‐affirming hormone therapy (GAHT) is not covered by insurance, so in practice, GAS is performed at the patient's expense.

In Japan, transgender individuals must meet five legal requirements to change their legal gender. Among these, two conditions—having no gonads or being in a permanent state of infertility (infertility requirement) and having an appearance similar to the genitalia of the desired gender (appearance requirement)—practically require undergoing GAS. This legal framework has sparked debate over the coercive nature of gender confirmation surgery. In October 2023, the Supreme Court ruled that the “infertility requirement” was unconstitutional and referred the “appearance requirement” back to the High Court for reconsideration. The Supreme Court's decision has the power to override legal provisions.^5^ Since then, clinical cases have been reported where gender change in official records has been recognized without undergoing gonadectomy. The profound impact of legal requirements on the decision‐making and lives of transgender individuals has come to light.

It remains unclear whether legal requirements, particularly the mandatory GAS for legal gender change, influence transgender individuals' decision‐making. Some individuals may choose this surgery with positive thoughts, whereas others may feel compelled to undergo it due to negative selection. This study was conducted to examine the significance of GAS for transgender individuals in Japan and to clarify whether the requirement for gonadectomy influences the decision to undergo GAS.

## SUBJECTS AND METHODS

This multicenter, prospective observational study was conducted at eight hospitals and clinics in Japan that provide GI care, including specialists in psychiatry, urology, gynecology, and plastic surgery. Ethical approval was obtained at the central research facility (approval number 352‐172). Participants were recruited from among outpatients visiting the participating facilities between March 1 and June 30, 2024. Eligible individuals were 18 years or older, had received a formal diagnosis of GI by medical institutions, and had not undergone GAS. Exclusion criteria included those without a formal diagnosis of GI and those who had already undergone GAS or gonadectomy. The history of GAHT or mastectomy was not an exclusion. Baseline demographic and medical data were collected, including age, duration of GAHT, and history of mastectomy. Written informed consent was obtained from all participants in accordance with ethical guidelines. Participants were asked to complete a self‐administered questionnaire regarding their willingness to GAS (see Appendix). The questionnaire included the following items: (1) Whether the participant desired GAS in the future, (2) reasons for desiring or not desiring GAS, (3) types of procedures they wished to undergo (with separate options for individuals assigned female at birth [AFAB] and individuals assigned male at birth [AMAB]), and (4) whether they would desire GAS, including gonadectomy, even if it was not legally required for a gender change. Most questions used multiple‐choice formats, and free‐text responses were permitted.

Descriptive statistics were used for continuous variables (mean, standard deviation, median, and range). For categorical variables, Fisher's exact test or the chi‐square test was applied to compare AFAB and AMAB participants. A *p*‐value of <0.05 was considered statistically significant.

## RESULTS

### Patient characteristics

A total of 109 individuals participated in the study. Of these, one case was excluded due to incomplete responses, and another was excluded due to unclear gender identity, leaving 107 cases (82 AFAB and 25 AMAB) for analysis. The median age was 31 years (range: 19–61), and 106 participants had already initiated GAHT. Among AFAB participants, 50 individuals (60.9%) had undergone mastectomy. The median duration of GAHT was 48 months (range: 4–300 months).

### Desire for GAS and reasons

When asked whether they wished to undergo GAS in the future, 74 participants (69%) responded “yes,” 31 (29%) responded “no,” and 2 (2%) responded “both” (interpreted as undecided).

The most frequently selected reason for desiring GAS was “to change legal gender (positive feeling)” (46 participants), followed by “to change legal gender (unavoidable)” (18 participants). Other reasons included “to achieve mental stability” (42), “to reduce gender dysphoria” (40), “to become my true self” (37), “discomfort with one's external genitalia” (35), “gonadectomy is desired (the gonads are unnecessary)” (35), and “at partner's request” (3).

**Figure 1:**
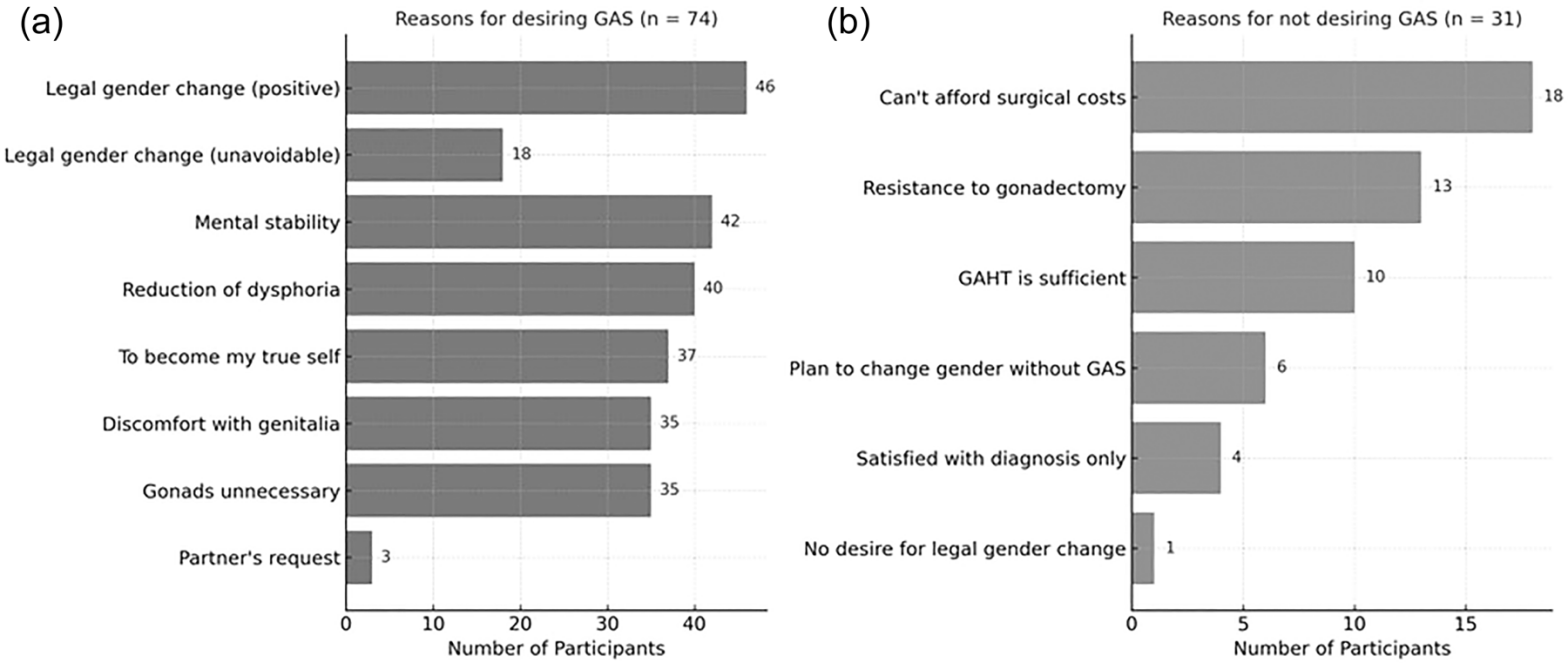
Reasons for desiring and not desiring gender‐affirming surgery (GAS). Participants (*n* = 107). (a) Reasons for desiring a GAS (*n* = 74) and (b) reasons for not desiring GAS (*n* = 31). GAHT, gender‐affirming hormone therapy.

Reasons for not desiring GAS included “unable to afford surgical costs at this time” (18 participants), “resistance to gonadectomy” (13), and “satisfaction with GAHT alone” (10). Based on the free description, six individuals indicated they planned to proceed with a legal gender change without undergoing surgery (Figure 1a,b).

### AFAB participants

**Figure 2:**
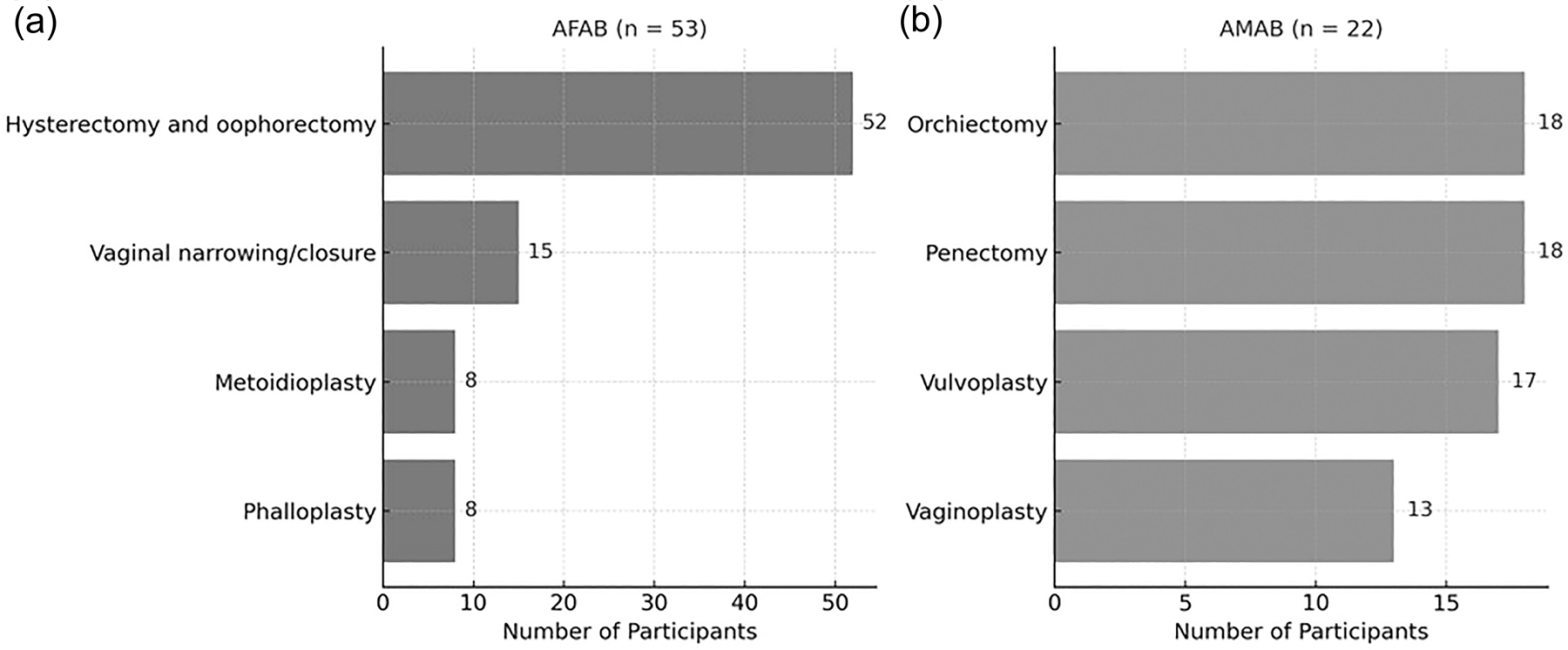
Desired surgical procedures among assigned female at birth (AFAB) and assigned male at birth (AMAB) participants. (a) AFAB respondents (*n* = 53) most requested hysterectomies and oophorectomies. (b) AMAB respondents (*n* = 22) equally requested orchiectomy, penectomy, and vulvoplasty.

Among the 82 AFAB participants, 52 (63%) expressed a desire for GAS, 28 (34%) did not, and 2 (3%) remained undecided. No significant difference in the willingness to GAS was found between those who had undergone mastectomy and those who had not (*p* = 0.69). Of the 53 who selected their desired procedures (multiple responses allowed), the most requested was for hysterectomy and oophorectomy (52), followed by vaginal narrowing or closure (15), metoidioplasty (8), and phalloplasty using a skin flap (8) (Figure 2a). One participant desired phalloplasty without undergoing a gonadectomy. Reasons for choosing surgery included: “The uterus and ovaries are unnecessary” (40), “to stop menstruation” (26), “the vagina is unnecessary” (14), “having a penis signifies being male” (6), “to void while standing” (5), and “to prevent gynecological diseases” (1).

Among the 26 who wished for hysterectomy and oophorectomy only, the most cited reasons were “concerns about the risk of surgical complications” (25), “possible change in surgical costs” (9), and “easier management of GAHT” (3). Based on the free description, other reasons included “requirement for legal gender change” (3), “desire to return to work early” (1), “family opposition” (1), and “satisfaction with the use of an epithesis” (1).

### AMAB participants

Among the 25 AMAB participants, 22 expressed a desire for GAS, whereas 3 did not. The most frequently chosen procedures were orchiectomy (18), penectomy (18), valvuloplasty (17), and vaginoplasty (13) (Figure 2b). Reasons for selecting these procedures included: a “desire for female genitalia” (17), believing that “gonads are unnecessary” (15), thinking “the penis is unnecessary” (13), wishing “to stop erections” (13), and a “desire to have sexual intercourse via the vagina” (6). Some wanted vaginal reconstruction even though they did not seek sexual intercourse, aiming to resemble cisgender women. One requested only an orchiectomy, citing “the risk of surgical complications” as the primary reason. Reasons for not opting for vaginoplasty included: “the risk of surgical complications” (5), “possible changes in surgical costs” (5), feeling “no need for sexual intercourse” (3), and “difficulty with vaginal dilation” (1).

### If gonadectomy is no longer a legal requirement

**Figure 3:**
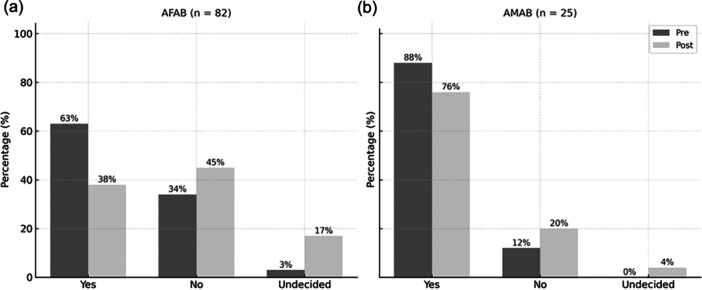
Gonadectomy desire before and after legal requirement removal comparison of percentages of (a) assigned female at birth (AFAB) (*n* = 82) and (b) assigned male at birth (AMAB) (*n* = 25) participants who desired gonadectomy before and after the legal requirement removal. Gray bars represent “pre‐infertility requirement removal (Pre),” and light gray bars represent “post‐infertility requirement removal (Post).”

When asked whether they would undergo GAS if a legal gender change were possible without gonadectomy, 50 participants (47%) answered “yes,” 42 (39%) answered “no,” and 15 (14%) did not respond (interpreted as undecided). Among AFAB participants (*n* = 82), 31 (38%) said “yes,” 37 (45%) said “no,” and 14 (17%) did not respond. Among AMAB participants (*n* = 25), 19 (76%) answered “yes,” 5 (20%) answered “no,” and 1 (4%) did not respond (Figure 3a,b).

## DISCUSSION

The method of determining legal gender is a critical issue closely tied to an individual's core identity and serves as the foundation for exercising rights in society. The requirements for changing legal gender can be broadly categorized into two types: those requiring medical intervention and those based on self‐determination.^6^ Among the medical intervention requirements, the infertility requirement (gonadectomy) has been a subject of international debate since its introduction in Sweden in 1972.^7^ Some argue that transgender individuals inherently seek sterilization as part of their gender transition.^8^ On the other hand, there are claims that mandatory sterilization constitutes a human rights violation. The “Yogyakarta Principles,” adopted in 2006 by an expert meeting on international human rights law, explicitly state that legal gender recognition should not require sterilization or other medical interventions, and that forced sterilization constitutes a human rights violation. Legal gender should be based on self‐determination.^9^ In a joint statement issued in 2014 by the World Health Organization and other organizations, the forced sterilization of individuals is also criticized.^10^, ^11^ Furthermore, in 2017, the European Court of Human Rights ruled that surgery or medical interventions (including sterilization) against the will of the individual violate Article 8 of the European Convention on Human Rights.^12^ Such decisions have prompted legislative reforms in many countries, with infertility requirements being abolished in Sweden (2013) and the Netherlands (2013), among others. The United Kingdom (2004) and Spain (2007) recently enacted laws on gender reassignment, but these do not include this requirement. On the other hand, Denmark (2014), Malta (2015), France (2016), and Finland (2023) have abolished both medical diagnosis and infertility requirements and adopted a self‐determination model.^6^, ^8^, ^12^, ^13^, ^14^

In Japan, the Act on Special Cases in Handling Gender Status for Persons with Gender Identity Disorder was enacted in 2003 and implemented in 2004 to facilitate legal gender change. This law required medical interventions, including infertility and appearance requirements. However, in October 2023, the Supreme Court finally ruled that the infertility requirement was unconstitutional.^5^ Since this landmark decision, the number of individuals permitted to change their legal gender without undergoing gonadectomy has increased. Meanwhile, the appearance requirement remained subject to ongoing review but was deemed potentially unconstitutional by a High Court in July 2024. A final ruling by the Supreme Court is now pending.

The findings of this study are noteworthy. At the time of the survey, 69% of respondents expressed a desire for GAS, including gonadectomy. Interestingly, the most commonly cited motivation was that legal gender change was perceived as a “positive” and meaningful goal. Only a minority described the procedure as “unavoidable,” suggesting that many view GAS in affirming, rather than obligatory, terms. On the other hand, the leading reason for not undergoing GAS was the financial burden. Although GAS procedures are technically covered by health insurance in Japan, most transgender individuals pay out of pocket for hormone therapy, and the system of “mixed treatment” effectively limits access to insurance coverage for surgical procedures. If GAHT were fully covered by insurance, the proportion of individuals pursuing GAS might increase, as financial barriers seem to outweigh physical and psychological considerations. However, when asked whether they would still undergo GAS if gonadectomy were no longer legally required, only 47% responded affirmatively. A closer look reveals that the proportion of AFAB individuals wishing to avoid gonadectomy increased significantly. In contrast, AMAB individuals largely continued to express a desire for GAS that includes gonadectomy. The increased number of undecided respondents may reflect internal conflict or transitional psychological states.

AFAB individuals usually experience clitoral enlargement that resembles a glans as a result of testosterone therapy. Additional masculinizing physical changes can occur due to GAHT.^15^ Since the ovaries are situated inside the abdominal cavity, the presence or absence of gonads is not visible externally. Consequently, some individuals may believe that a legal gender change can be achieved without gonadectomy or genital surgery. However, retaining the uterus or ovaries raises concerns about the potential return of menstruation if GAHT is discontinued. Furthermore, some individuals may experience psychological discomfort with the presence of a vagina. In contrast, AMAB individuals typically do not undergo significant atrophy of the penis or testis with estrogen therapy alone. Many participants in this study report seeking GAS not merely for hormonal or medical reasons but due to a profound discomfort with their external genitalia and a desire to become their “true selves.” As a result, AMAB individuals are more likely to pursue full genital reconstruction rather than limit surgery to orchiectomy. This indicates that appearance plays a crucial role for AMAB individuals in the context of gender affirmation. While some concerns have been raised about the social implications of allowing legal gender change without gonadectomy, the present study suggests that many individuals still view genital surgery as personally necessary.

### Limitations

This study is marked by nearly all participants having already undergone GAHT and a low proportion of AMAB individuals. This demographic bias may introduce selection bias. In particular, the small number of AMAB participants may make it difficult to generalize the results to AMAB individuals. Additionally, the use of self‐administered questionnaires could have introduced anchoring bias, potentially influencing participants' responses. Despite these limitations, this study offers valuable insights into the perspectives of transgender individuals in Japan regarding GAS and gonadectomy. Notably, this is the first study of its kind conducted in Japan and the first multicenter prospective study in this area. It is also a timely study conducted shortly after the Supreme Court ruled that the infertility requirement should be removed for legal gender recognition. As legal requirements regarding gender recognition continue to evolve, further research is necessary to evaluate the actual impact on decision‐making and access to medical care.

## CONCLUSION

Amendments to Japan's legal requirements for gender recognition—particularly the abolition of the infertility requirement (gonadectomy)—have the potential to reduce the number of individuals compelled to undergo surgery. However, the fact that a certain percentage of GI individuals still wish for GAS highlights the importance of maintaining access to this surgery even within a more flexible legal framework. Conversely, the primary reason for not desiring GAS is the difficulty in covering costs, which is likely to ignite new discussions regarding the management of medical expenses for transgender physical treatments, particularly GAHT.

## AUTHOR CONTRIBUTIONS

Koji Ichihara conceived the study, and Koji Ichihara and Wakako Yorozuya designed the study. Nodoka Kozen, Teruo Abe, Manabu Nakagawa, Koji Niwa, Tsuyoshi Baba, Utako Ikeda, Satoshi Nishikawa, and Daito Nishiyama collected questionnaire data and clinical information. Koji Ichihara and Wakako Yorozuya analyzed the results. Koji Ichihara and Wakako Yorozuya wrote the manuscript together with Naoya Masumori. All authors discussed the results and approved the final manuscript.

## CONFLICT OF INTEREST STATEMENT

The authors declare no conflicts of interest.

## ETHICS APPROVAL STATEMENT

This study was approved by the Ethics Review Committee of Sapporo Medical University (approval number 352‐172).

## PATIENT CONSENT STATEMENT

Written informed consent was obtained from all participants in the study.

## CLINICAL TRIAL REGISTRATION

N/A.

## Data Availability

The data that support the findings of this study are available from the corresponding author upon reasonable request. However, the data are not publicly available due to considerations of participant privacy and ethical restrictions.

## Appendix

Questionnaire on Gender‐Affirming Surgery

**1. Do you wish to undergo gender‐affirming surgery?**

□ Yes to → (2‐2)
□ No to → (2‐1)

**2‐1. If “no,” circle all the reasons that apply.**

A) Enough to confirm the diagnosis
B) Satisfaction with gender‐affirming hormonal therapy alone
C) Unable to afford surgical costs at this time
D) Don't want to change my legal gender
E) Resistance to gonadectomy
F) Other (free description): ___________

**2‐2. If “yes,” circle all the reasons that apply.**

(What do you desire from gender‐affirming surgery?)

A) To achieve mental stability
B) To reduce gender dysphoria
C) To become my true self
D) Discomfort with one's external genitalia
E) Gonadectomy is desired (the gonads are unnecessary)
F) At the partner's request
G) To change legal gender (positive feeling)
H) To change legal gender (unavoidable)
I) Other (free description): ___________

**3. If “yes,” circle all the desires for surgery that apply.**

**3‐1‐1. If you are a transgender male, please select from here.**

A) Hysterectomy and oophorectomy
B) Vaginal narrowing or closure
C) Metoidioplasty
D) Phalloplasty using a skin flap

**3‐1‐2. Why did you choose the surgery?**

A) Uterus and ovaries are unnecessary
B) The vagina is unnecessary
C) To stop menstruation
D) Having a penis signifies being male
E) To void while standing
F) Other (free description): ___________

**3‐1‐3. If you choose only “hysterectomy and oophorectomy,” please explain your reasoning.**

A) Concerns about the risk of surgical complications
B) Possible changes in surgical costs
C) Easier gender‐affirming hormonal therapy management
D) Other (free description): ___________

**3‐2‐1. If you are a transgender woman, please select from here.**

A) Orchiectomy
B) Penectomy
C) Vulvoplasty
D) Vaginoplasty

**3‐2‐2 Why did you choose the surgery?**

A) The testes are unnecessary
B) The penis is unnecessary
C) Desire to stop erections
D) Desire for female genitalia
E) Desire to have sexual intercourse via the vagina
F) Other (free description): ___________

**3‐2‐3 If you choose only “orchiectomy,” please explain your reasoning.**

A) Concerns about the risk of surgical complications
B) Possible changes in surgical costs
C) Easier gender‐affirming hormonal therapy management
D) Other (free description): ___________

**3‐2‐4 If you did not wish to have “vaginoplasty,” please explain your reasoning.**

A) Concerns about the risk of surgical complications
B) Possible changes in surgical costs
C) No need for sexual intercourse
D) Have sexual intercourse not via the vagina (e.g, via the anus)
E) Other (free description): ___________

**4. The Supreme Court ruled that the surgery requirement of the Gender Identity Disorder Special Cases Act, “the absence of gonads or permanent absence of gonadal function,” is unconstitutional. If it were possible to change your legal gender without gonadectomy, do you wish to undergo gender‐affirming surgery? Note that no ruling was made on the appearance requirements (penectomy and vulvar reconstruction), and the case was remanded to the High Court.**

A) Yes
B) No
